# Genomic Characterization of *Yersinia enterocolitica* Isolates, Costa Rica

**DOI:** 10.3201/eid3104.240963

**Published:** 2025-04

**Authors:** Cyril Savin, Gletty Oropeza, Luis A. Barboza Fallas, Olga Rivas-Solano, Grettel Chanto, Javier Pizarro-Cerdá

**Affiliations:** Institut Pasteur, Paris, France (C. Savin, J. Pizarro-Cerdá); Instituto Costarricense de Investigación y Ensenanza en Nutricion y Salud, Cartago, Costa Rica (G. Oropeza, G. Chanto); Instituto Tecnológico de Costa Rica, Cartago (L.A. Barboza Fallas, O. Rivas-Solano)

**Keywords:** Yersinia enterocolitica, bacteria, zoonoses, food safety, enteric infections, genomic surveillance, bacterial cluster, antimicrobial resistance, Costa Rica

## Abstract

Data on enteric yersinioses in Central America are limited. Genomic characterization of 78 *Yersinia* *enterocolitica* isolates from Costa Rica indicated persistent infection-source circulation between animal reservoirs and humans, as well as unusual antimicrobial resistance levels. Our study highlights the importance of genomic surveillance to monitor *Yersinia*-caused infections in Costa Rica.

The *Yersinia* genus encompasses 2 enteropathogenic species, *Y. enterocolitica* and *Y. pseudotuberculosis* (*1*,*2*). Those bacteria are the cause of foodborne infections that range from mild enteritis, especially in children, to systemic infections in the elderly or patients with underlying disorders (*3*). Enteric yersiniosis is the third most frequently reported zoonosis in Europe, mainly caused by *Y. enterocolitica* infections (*4*). Isolates can be classified into nonpathogenic genotypes (1Aa and 1Ab) and 11 other pathogenic genotypes (*2*).

In France, genomic surveillance based on a *Y. enterocolitica* core genome multilocus sequence typing (cgMLST) scheme was useful in identifying genetically closely related isolates and initiating an epidemiologic investigation with public health authorities to identify a common source of infection (*5*). Epidemiologic information on enteric yersinioses is scarce in the Americas. We studied *Yersinia* isolates from Costa Rica to evaluate their circulation in the country and to characterize them at the genomic level. 

We analyzed 78 isolates collected during 2003–2023; all were of clinical origin except 1 veterinary isolate (Appendix 1). Genomic characterization based on a *Yersinia* 500-gene cgMLST enabled us to identify all isolates as pathogenic *Y. enterocolitica*: 2 isolates belonged to genotype 2/3-5a and the 76 others to genotype 4. All patients (n = 77) experienced enteric infections; we only identified pathogenic isolates. Epidemiologic records of the 77 clinical isolates showed that the sex ratio was 1:1 as expected in other countries (*4*). Most (76%) of the isolates were collected from patients <20 years of age; 20% were from adults 21–60 years of age. We observed a slight (4%) increase in patients >61 years of age. Most (97%) isolates came from feces, indicating that patients had a digestive form of infection, whereas 3% of the isolates were collected from blood (Appendix 2 Table). Overall, the epidemiology of *Y. enterocolitica* isolates circulating in Costa Rica was similar to that of other countries (*4*).

Geographic distribution of the isolates showed that the 78 isolates were distributed in the 7 provinces of Costa Rica; 60 (77%) isolates were from the Gran Area Metropolitana, a densely populated region around San Jose, the capital city (Figure). Phylogenetic reconstruction of the 76 genotype 4 isolates revealed 4 distinct bacterial subgroups (Figure). No geographic association was found between subgroups or provinces. We conducted a genomic comparison of isolates on the basis of a *Y. enterocolitica* 1,727-gene cgMLST and subsequent clustering of isolates with a 5-mismatch threshold; we found that 51/76 isolates belonged to 9 clusters of >2 isolates (Table; Appendix 1 Figure). We identified 3 clusters with isolates collected over a long period: cluster 6122 for 10 years, cluster 6127 for 13 years, and cluster 6124 for 8 years. Those periods suggest persistence of the source of contamination. We found that cluster 3151 is composed of mixed clinical and veterinary isolates, suggesting circulation between the animal reservoir and humans (Table; Appendix 2 Table). Of note, cluster 5652 is composed of 2 isolates from Costa Rica and 1 from France. Epidemiologic records of the France isolate showed that the patient was returning from Costa Rica at the time of sampling, suggesting that he was infected during his trip.

**Figure:**
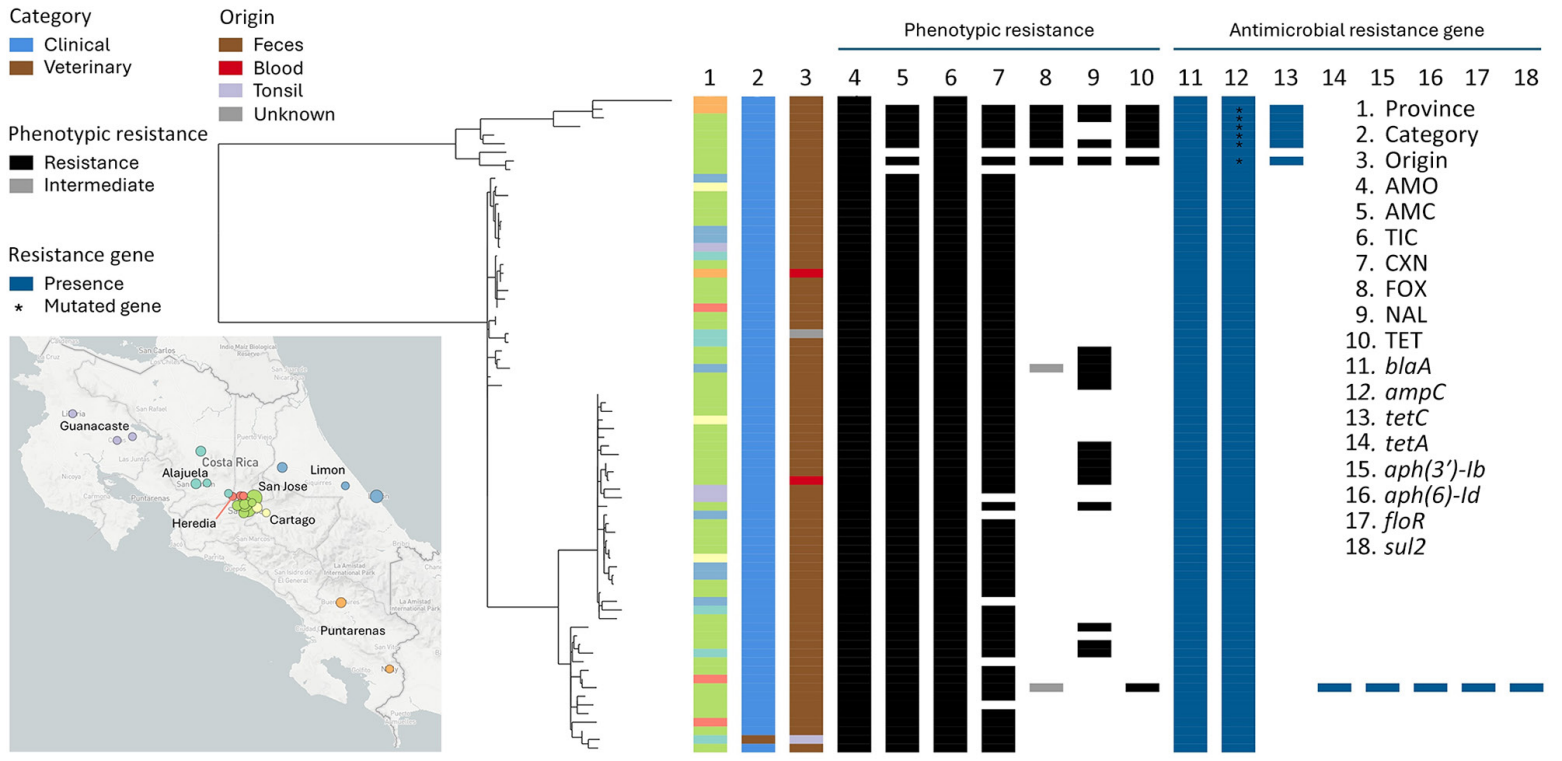
Phylogenetic tree of 76 *Yersinia enterocolitica* genotype 4 isolates collected in Costa Rica, 2003–2023. The tree was reconstructed with IQ-TREE2 (http://www.iqtree.org) after alignment of the 1,727 genes of the cgMLST *Y. enterocolitica*. Columns show information on province (1), category (2), origin (3), antimicrobial resistance by drug (4–10), and by gene (11–18). Map (inset) shows cases by province. AMC, amoxicillin/clavulanic acid; AMO, amoxicillin; cgMLST, core genome multilocus sequence typing; CXN, cefalexin; FOX, cefoxitin; NAL, nalidixic acid; TET, tetracycline; TIC, ticarcillin.

**Table:** Epidemiologic characteristics of cgMLST clusters of *Yersinia enterocolitica* isolates, Costa Rica*

cgMLST cluster	No. isolates from Costa Rica	No. isolates in cluster	Category	Country	Isolation dates
6122	27	27	Clinical	Costa Rica	2004–2014
6127	8	8	Clinical	Costa Rica	2006–2019
6180	3	3	Clinical	Costa Rica	2014–2016
3151	3	3	Clinical, veterinary	Costa Rica	2018–2022
6124	2	2	Clinical	Costa Rica	2005–2013
6149	2	2	Clinical	Costa Rica	2007–2008
6164	2	2	Clinical	Costa Rica	2008–2011
6212	2	2	Clinical	Costa Rica	2012–2015
5652	2	3	Clinical	Costa Rica and France	2015–2023

*Clinical isolates are from human samples. cgMLST, core genome multilocus sequence typing.

Among the 76 genotype 4 isolates, antimicrobial susceptibility testing revealed unusually high levels of resistance to cefoxitin (7.9%), nalidixic acid (23.7%), and tetracycline (9.2%) (Figure; Appendix 1 Table) (*6*). Nalidixic acid resistance was associated with mutations in the quinolone resistance–determining region of the *gyrA* gene; we identified the previously described S83R mutation (*7*) in 15 isolates and D87G mutation in 3 isolates. Cefoxitin resistance in 6 isolates was associated with a G751A mutation of the *ampC* gene, identical to the mutation present in the naturally cefoxitin-resistant genotype 2/3-5a. Those 6 isolates are in the upper part of the tree, suggesting the mutation was acquired during emergence of the branch (Figure). In 6/7 tetracycline-resistant isolates, resistance was associated with acquisition of the *tetC* gene located on a 13.7 kb plasmid circulating in other enterobacteria. Those 6 isolates are on the upper branch of the tree, suggesting that plasmid acquisition occurred during emergence of the branch (Figure). The other tetracycline-resistant isolate was associated with the *tetA* gene, together with *aph(3ʹ)-Ib* and *aph(*6*)-Id*, *floR*, and *sul2*, located on an 81.2 kb multidrug-resistant plasmid (Figure). The presence of those genes was phenotypically associated with resistance to tetracycline, streptomycin, chloramphenicol, and sulfonamides.

In summary, this study brings insights into the distribution of enteropathogenic *Yersinia* species circulating in Costa Rica. Diversity is low because most of the *Y. enterocolitica* isolates belonged to biotype 4. Many closely related isolates were identified within a long timeframe, sometimes between human clinical and veterinary isolates, suggesting a persistence of the source of infection and the circulation between the reservoir and humans. Implementation of routine genomic surveillance in Costa Rica could help to monitor the circulation of closely related *Yersinia* isolates and initiate epidemiologic investigation to implement control measures in the event of an outbreak.

Appendix 1 Additional information about genomic characterization of *Yersinia enterocolitica* isolates from Costa Rica.

Appendix 2 Metadata for 78 isolates of Y. enterocolitica isolates from Costa Rica.
