# Supplementary material for: Genomic Characterization of Yersinia enterocolitica Isolates, Costa Rica
Source: Emerg Infect Dis. 2025 Apr;31(4):848–60. doi: 10.3201/eid3104.240963 (PMC11950257; doi:10.3201/eid3104.240963)
Supplement: Appendix 1 — Additional information about genomic characterization of Yersinia enterocolitica isolates from Costa Rica. [file 24-0963-Techapp-s1.pdf]

DOI: https://doi.org/10.3201/eid3104.240963

EID cannot ensure accessibility for supplementary materials supplied by authors. Readers who have difficulty accessing supplementary content should contact the authors for assistance.

Genomic Characterization of *Yersinia enterocolitica* Isolates, Costa Rica

Appendix 1

Materials and Methods

Bacterial Isolation

This study includes 78 isolates collected between 2003 and 2023 in the 7 provinces of Costa Rica. The 77 clinical isolates were obtained by the Institute for Research and Teaching in Nutrition and Health (INCIENSA) in the context of public health surveillance of enteric diseases. Direct culturing without enrichment allowed to recover isolates from patients presenting various symptoms, mostly acute diarrhea. A total of 77 isolates from the stock culture collection of the National Reference Center of Bacteriology were taken from storage at −70°C and passaged on blood agar. Identification and susceptibility cards were inoculated according to the directions of the manufacturer and processed in the Vitek2 instrument. In addition, conventional biochemicals tests and miniaturized biochemical assays (API 20E) were performed.

The veterinary isolate, collected from a pig tonsil, was obtained by the Technological institute of Costa Rica, San Carlos campus, in the context of a research project related to food borne pathogens. Twenty-five g of pig tonsil was inoculated in 225 mL of peptone sorbitol bile (PSB) broth (Millipore, Burlington, Massachusetts, United States) and incubated at 4°C. Every 7 days, for a month, 1 mLof the culture was subcultured in 100 mLof PSTA Enrichment Broth Base (Himedia, Pennsylvania, United States) and incubated at 28°C for 48 hours. Then, 10 µl were streaked onto semi-solid Cefsulodin-Irgasan-Novobiocin Agar (CIN) (Yersinia Selective Agar Base and Yersinia Selective Supplement, Millipore, Burlington, Massachusetts, United States). As indicated in the ISO 10273:2017 method (*1*), *Y. enterocolitica* colonies on CIN agar are typically small and smooth, with a red center and a translucent rim, and when examined with obliquely transmitted light, they are non-iridescent and finely granular.

DNA Extraction and Genome Sequencing

Isolates were grown for 18 hours at 28°C in LB broth under agitation at 150 rpm. DNA extraction was performed with the Maxwell RSC Cultured Cells DNA kit (reference AS1620, Promega, Madison, USA) on a Maxwell CSC 48 Instrument (Promega, Madison, USA) according to the manufacturer's instructions. DNA quantity and purity were assessed using a Qubit fluorometer (Thermo FisherScientific, Waltham, MA, USA). Genomic libraries were prepared using the Nextera XT Sample Kit (Illumina, San Diego, CA, USA), and DNA sequencing was performed on a NextSeq 500 platform (Illumina) using 2× 150-bp paired-end runs. Raw data were assembled using the fq2dna pipeline (https://gitlab.pasteur.fr/GIPhy/fq2dna). To identify plasmids present in isolates IP51152 and IP51348, genomic DNA extraction was performed using a PureLink Genomic DNA mini kit (reference K182002, Invitrogen) following the manufacturer's instructions. Long-read genome sequencing was performed using Oxford Nanopore Technologies (ONT) with the Plasmidsaurus service (www.plasmidsaurus.com). Hybrid genome assembly using both Illumina and ONT sequencing data was performed as previously described (https://github.com/rrwick/Perfect-bacterial-genome-tutorial) (*2*).

Genomic Characterization and Molecular Typing

Taxonomic assignment of the isolates was performed using a 500-gene cgMLST Yersinia as previously described (*3*). Molecular typing of the isolates was performed using a 1,727-genes cgMLST *Y. enterocolitica* followed by clustering of the isolates with a maximum of 5 allelic differences as previously described (*4*). Phylogenetic reconstruction was carried out on the 1,727 genes of the cgMLST *Y. enterocolitica* scheme after their extraction using the "Export" plugin at https://bigsdb.pasteur.fr/yersinia. For each isolate, the genes were concatenated and then aligned using mafft v7.467 (*5*). From this alignment, a phylogenetic reconstruction was performed with IQ-TREE2 (*6*) using a bootstrap value of 1,000 and by first determining the best substitution model thanks to the "ModelFinder" implemented in IQ-TREE2.

Antimicrobial Susceptibility Testing and Genotypic Prediction of Antimicrobial Resistance

The antibiotic resistance profiles of the isolates were determined by disk diffusion method on Mueller-Hinton II agar according to the European Committee on Antimicrobial Susceptibility Testing (https://www.eucast.org) recommendation with an incubation temperature of 28°C. Antibiotics tested were amoxicillin (AMO – 20 µg), cefoxitin (FOX - 30 µg),

cefotaxime (COX – 5 µg), ceftazidime (CZD – 10 µg), ciprofloxacin (CIP – 5 µg), nalidixic acid (NAL – 30 µg), cefalexin (CXN – 30 µg), cotrimoxazole (Trimethoprim – Sulfamethoxazole, SXT 1.25 + 23.75 µg), amoxicillin + clavulanic acid (AMC, 20 µg + 10 µg), ticarcilline (TIC – 75 µg), tetracycline (TET – 30 µg), ertapenem (ETP – 10 µg), and amikacin (AKN - 30 µg). In addition, sulfonamides (SSS – 300 µg), chloramphenicol (CHL – 30 µg), and streptomycin (SMN – 10 µg) were tested for the multidrug resistant isolate IP51348.

The presence of antimicrobial resistance genes presence in the genomes was identified using the Resfinder Database version 4.3.3 (*7*).

Data Visualization

Epidemiologic, phenotypic and genetic data along with the phylogenetic tree were visualized using Microeact www.microreact.org (*8*). The cgMLST *Y. enterocolitica* profiles of the 76 *Y. enterocolitica* genotype 4 isolates were exported from the BIGSdb database using "Export plugin" at https://bigsdb.pasteur.fr/yersinia and the minimum spanning tree was built using BioNumerics v.7.6 (Applied-Maths, Sint-Martens-Latem, Belgium).

Data Availability

The cgMLST allelic profiles of the 78 isolates have been made publicly available in the *Yersinia* BIGSdb database (https://bigsdb.pasteur.fr/yersinia). Sequence data was made publicly available in NCBI/EBI/DDJJ databases (BioProject no. PRJNA1128560).

Supplementary References

1. International Organization for Standardization. ISO 10273:2017. Microbiology of food and animal feeding stuffs–horizontal method for the detection of presumptive pathogenic *Yersinia enterocolitica* [cited 2025 Feb 26]. https://www.iso.org/standard/63329.html

2. Wick RR, Judd LM, Holt KE. Assembling the perfect bacterial genome using Oxford Nanopore and Illumina sequencing. PLOS Comput Biol. 2023;19:e1010905. PubMed https://doi.org/10.1371/journal.pcbi.1010905

3. Savin C, Criscuolo A, Guglielmini J, Le Guern AS, Carniel E, Pizarro-Cerdá J, et al. Genus-wide *Yersinia* core-genome multilocus sequence typing for species identification and strain characterization. Microb Genom. 2019;5:e000301. PubMed https://doi.org/10.1099/mgen.0.000301

4. Le Guern AS, Savin C, Chereau F, Tessier S, Guglielmini J, Brémont S, et al. A novel cgMLST for genomic surveillance of *Yersinia enterocolitica* infections in France allowed the detection and investigation of outbreaks in 2017–2021. Microbiol Spectr. 2024;12:e0050424. PubMed https://doi.org/10.1128/spectrum.00504-24

5. Katoh K, Standley DM. MAFFT multiple sequence alignment software version 7: improvements in performance and usability. Mol Biol Evol. 2013;30:772–80. PubMed https://doi.org/10.1093/molbev/mst010

6. Minh BQ, Schmidt HA, Chernomor O, Schrempf D, Woodhams MD, von Haeseler A, et al. IQ-TREE 2: new models and efficient methods for phylogenetic inference in the genomic era. Mol Biol Evol. 2020;37:1530–4. PubMed https://doi.org/10.1093/molbev/msaa015

7. Bortolaia V, Kaas RS, Ruppe E, Roberts MC, Schwarz S, Cattoir V, et al. ResFinder 4.0 for predictions of phenotypes from genotypes. J Antimicrob Chemother. 2020;75:3491–500. PubMed https://doi.org/10.1093/jac/dkaa345

8. Argimón S, Abudahab K, Goater RJE, Fedosejev A, Bhai J, Glasner C, et al. Microreact: visualizing and sharing data for genomic epidemiology and phylogeography. Microb Genom. 2016;2:e000093. PubMed https://doi.org/10.1099/mgen.0.000093

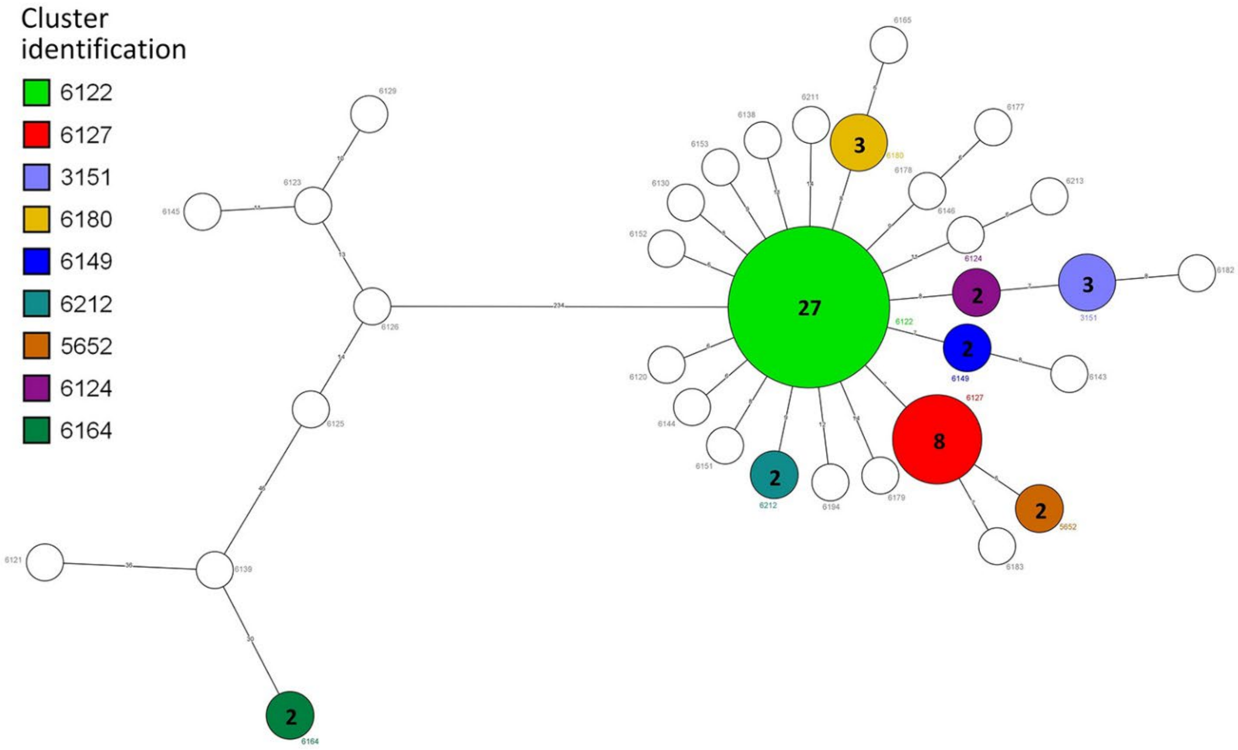

Appendix 1 Figure. Minimum spanning tree of the 76 *Yersina enterocolitica* genotype 4 isolates collected from 2003 to 2023 in Costa Rica. The tree was built based on the allelic profiles of the 1,727 genes cgMLST *Y. enterocolitica*. Circles represent the different profiles and sizes are proportional to the number of isolates within. All the allelic profiles with a maximum of 5 allelic distance were collapsed in the same circle. Branch lengths, reflecting the allelic differences between the profiles using square root scaling, are indicated on the branches. Cluster numbers are indicated close to each circle. When ≥2 isolates, the number of isolates present in a cluster are indicated within the circles.
